# Direct Effects of Light on Sleep under Ultradian Light-Dark Cycles Depend on Circadian Time and Pulses Duration

**DOI:** 10.3390/clockssleep4020019

**Published:** 2022-03-24

**Authors:** Fanny Fuchs, Ludivine Robin-Choteau, Laurence Hugueny, Dominique Ciocca, Patrice Bourgin

**Affiliations:** 1 Inovarion, 75005 Paris, France; fanny.fuchs@inovarion.com; 2 Institut des Neurosciences Cellulaires et Intégratives—Centre National de la Recherche Scientifique (CNRS), UPR3212, Université de Strasbourg, 67000 Strasbourg, France; l.choteau@ceed-diabete.org; 3 Centre Européen du Diabète, 67200 Strasbourg, France; 4 CHRU Strasbourg—Centre des Troubles du Sommeil, 67000 Strasbourg, France; laurence.hugueny@chru-strasbourg.fr; 5 Chronobiotron—UMS3415, Centre National de la Recherche Scientifique (CNRS), Université de Strasbourg, 67000 Strasbourg, France; ciocca@neuro-cnrs.unistra.fr

**Keywords:** sleep and waking, photic regulation, mice, alertness, sleep homeostasis, slow wave activity, ECoG power spectrum

## Abstract

Ultradian light–dark cycles in rodents are a precious tool to study the direct effects of repeated light exposures on sleep, in order to better understand the underlying mechanisms. This study aims to precisely evaluate the effects of light and dark exposures, according to circadian time, on sleep and waking distribution and quality, and to determine if these effects depend on the duration of light and dark pulses. To do this, mice were exposed to 24 h-long ultradian light–dark cycles with different durations of pulses: T2 cycle (1 h of light/1 h of dark) and T7 cycle (3.5 h of light/3.5 h of dark). Exposure to light not only promotes NREM and REM sleep and inhibits wake, but also drastically alters alertness and modifies sleep depth. These effects are modulated by circadian time, appearing especially during early subjective night, and their kinetics is highly dependent on the duration of pulses, suggesting that in the case of pulses of longer duration, the homeostatic process could overtake light direct influence for shaping sleep and waking distribution.

## 1. Introduction

In 1982, Borbély described sleep regulation as a two-process model, relying on a reciprocal interaction between a process controlled by the circadian clock and a homeostatic process, and this model has been prominent in the sleep field in the past decades [1,2,3]. Light also exerts powerful effects on sleep and waking in different ways. First of all, the light/dark alternation is a key Zeitgeber that drives the circadian timing system, which in turns acts to time sleep and wakefulness to the 24 h day [4]. Moreover, several papers have more recently highlighted the importance of light direct effects, independent of the circadian system, on sleep, waking and behavior [5,6,7]. These data obtained in rodents led us to propose a three-process model including the direct photic influence in addition to the homeostatic and circadian drives [6,8] and ongoing studies are underway in order to validate this model in humans.

Ultradian light–dark (LD) cycles, consisting of the alternation of short light and dark exposures in rodents, are a precious tool to study the influence of light and dark exposure, according to circadian time, on the distribution and quality of sleep and waking. Whereas it is now clearly demonstrated that light or dark exposure influence sleep and waking [6,9,10,11,12], the effects of such ultradian LD cycles on the distribution of the different vigilance states remain controversial [5,13,14,15,16,17,18] and their influence on sleep and wake quality has been poorly explored [5,13]. The three-process model suggests that the direct effects of light interact with the circadian and homeostatic regulations of sleep under such ultradian cycles. Therefore, the aims of this study are (1) to precisely evaluate the effects of light and dark exposures, according to circadian time, on sleep and waking distribution and quality, and (2) to determine if these effects depend on the duration of LD cycles. To do this, mice were exposed during 24 h to two ultradian LD cycles of different durations: T2 cycle, consisting of the alternation of 1 h of light/1 h of dark, and T7 cycle, consisting of 3.5 h of light/3.5 h of darkness. Exposure to light promotes both NREM and REM sleep and induces a decrease in wake amounts and alertness. These effects are modulated by circadian time, appearing especially during early subjective night, and their kinetics is highly dependent on the duration of pulses.

## 2. Results

### 2.1. The Direct Effects of Light and Dark Pulses on Sleep and Waking Depend on Circadian Time

**Figure 1:**
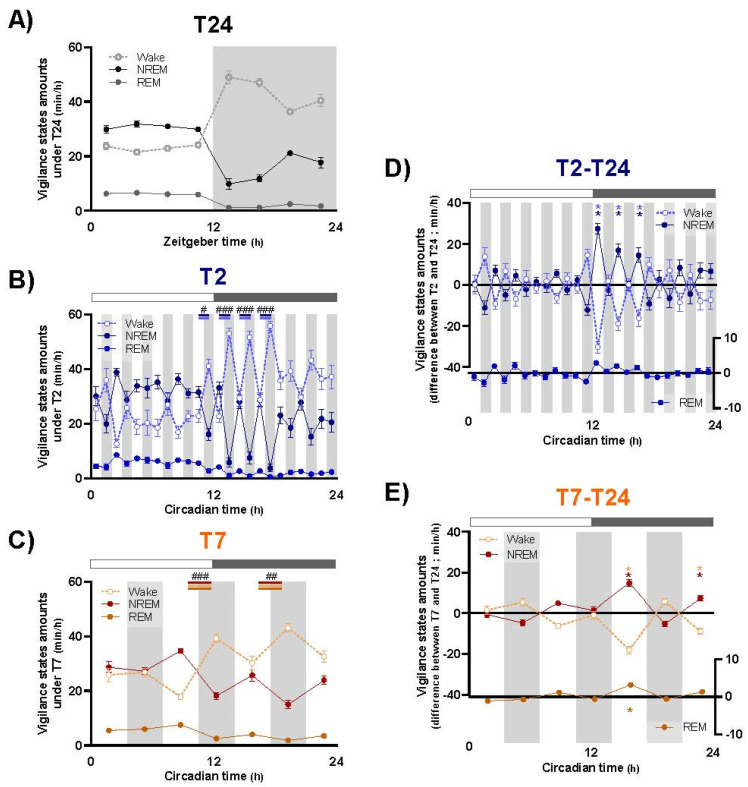
Influence of T2 and T7 ultradian light–dark cycles on wake, NREM and REM sleep distribution. Time course of wake (pale dotted lines), NREM sleep (dark solid lines) and REM sleep (pale solid lines) amounts across T24 ((**A**) per 3 h bins), T2 ((**B**) per 1 h bins) and T7 ((**C**) per 3.5 h bins) LD cycles. Difference of wake, NREM and REM sleep amounts between each light and dark pulse of T2 (**D**) or T7 (**E**) cycles and corresponding time points under T24 cycle. Circles represent means ± SEM. Grey areas delineate dark exposures. #, *p* < 0.05, ##, *p* < 0.01, ###, *p* < 0.001: difference between consecutive light and dark pulses; *, *p* < 0.05: difference between results obtained under T2/T7 and T24 cycle for specific light pulses. T24: *n* = 10; T2: *n* = 9; T7: *n* = 7.

The direct effects of 1 h (T2 cycle) and 3.5 h (T7 cycle) light and dark pulses on the distribution of wake, NREM and REM sleep amounts are represented in Figure 1, with baseline data under T24 in Figure 1A.

Overall, light pulses drastically promote NREM sleep and have lesser effects on REM sleep, but those effects are strongly dependent on circadian time. Indeed, this photic regulation of sleep is more pronounced during the subjective dark period (nighttime) than during the subjective light period (daytime). The T2 cycle, because of shorter pulses, allows researchers to analyze the influence of circadian time on the magnitude of light effects with greater time accuracy. Under the T2 cycle, the greatest effects of alternating 1 h light and dark pulses on sleep amounts appear during the early subjective night, from CT12 to CT18 (Figure 1B; Supplementary Table S1), as illustrated by the difference calculated for each of the light or dark pulse between T2 and T24 (calculation made for same 1 h time points, Figure 1D; Supplementary Table S2). During the subjective day, dark exposures slightly promote wakefulness, primarily for the first (ZT1) and last (ZT11) pulses, whereas photic regulation of sleep and wake is at a very low level for the rest of the period. Exposure to longer light and dark pulses (3.5 h) under the T7 cycle significantly modifies wake, NREM and REM sleep distribution, an effect observed from CT7 to CT21 (Figure 1C; Supplementary Table S1). The 3.5 h light pulses promote NREM and REM sleep, mainly during the subjective night, whereas dark pulses enhance wake (Figure 1E; Supplementary Table S2). Remarkably, the main effect of light is observed during early subjective night, for the same circadian times as T2 cycle, the increase in both NREM and REM sleep amounts being significantly higher between CT14 and CT17.5 than the one observed between CT21 and CT24 (ANOVA, effect of time: NREM: F_(6,36)_ = 20.70; *p* < 0.001; REM: F_(6,36)_ = 15.82; *p* < 0.001; Tukey: *p* < 0.05 for NREM and REM sleep).

### 2.2. Light Direct Effects on Sleep Result Mainly from an Increase in the Number of NREM and REM Sleep Episodes, Whereas Wake Inhibition Is Accompanied by a Drastic Fragmentation of Its Episodes

**Figure 2:**
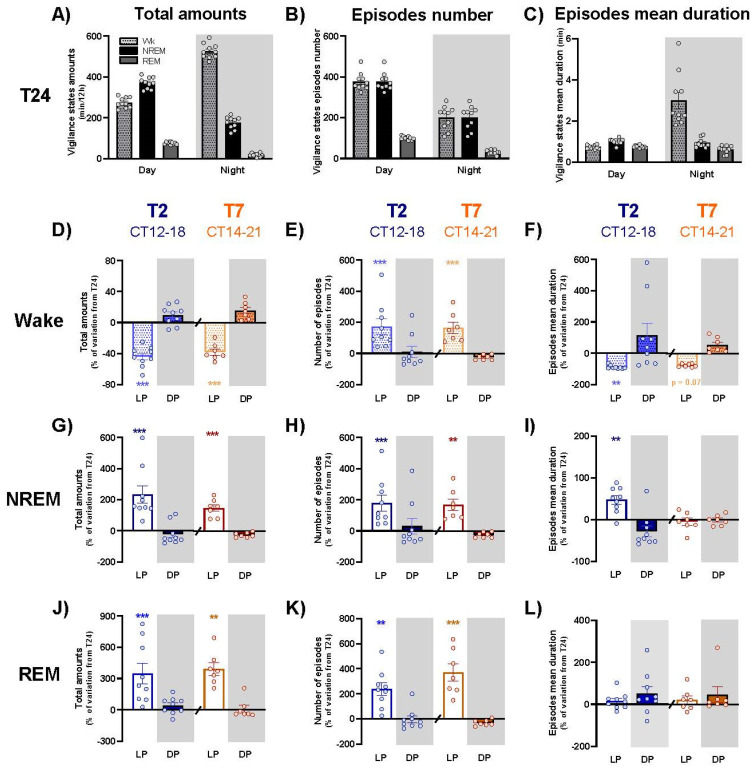
Influence of light and dark pulses on wake, NREM and REM sleep organization under T2 and T7, compared to T24 cycle. Wake (pale hatched bars), NREM (dark bars) and REM (pale bars) sleep total amounts (**A**), number of episodes (**B**) and episodes’ mean duration (**C**) under day and night of T24 cycle. The percentage of variation from T24 cycle for total amounts of wake (**D**), NREM (**G**) and REM (**J**) sleep, number of episodes of wake (**E**), NREM (**H**) and REM (**K**) sleep, and for episodes mean duration of wake (**F**), NREM (**I**) and REM (**L**) sleep during light (empty bars) and dark (filled bars) pulses averaged for CT12-18 under T2 (blue) and CT14-21 under T7 cycle (orange). Histograms represent means ± SEM, circles represent individual values. Grey areas delineate dark exposures. **, *p* < 0.01, ***, *p* < 0.001: difference between results obtained under T2/T7 and T24 cycle. LP: light pulse; DP: dark pulse. T24: *n* = 10; T2: *n* = 9; T7: *n* = 7.

In order to further understand the effects of the T2 and T7 cycles on sleep and waking organization, we calculated for each vigilance state the amount, number of episodes and their mean duration for each of the light and dark pulses, and we compared the values to those obtained under baseline T24. Baseline data under T24 are represented in Figure 2A–C.

During subjective night of both the T2 (CT12-18) and T7 (CT14-21) cycles, the light-induced decrease in wake amounts described earlier (Figure 1D,E) is due to a dramatic decrease in the episodes’ mean duration (Figure 2D,F; Supplementary Table S3), in spite of an increase of almost 200% of their number (Figure 2E; Supplementary Table S3). The opposite, but weaker, effect is observed for dark exposures during subjective day (Supplementary Figure S2A–C; Supplementary Table S4). Given the large range of wake episodes duration (from a few seconds to several hours), mean episodes’ duration is not the most representative way to illustrate T2 and T7 cycle effect on wake fragmentation. To do this, we computed time-weighted frequency histograms in order to evaluate the influence of the T2 cycle on wake fragmentation (Supplementary Figure S1). Thus, we could observe that under T24, total wake amounts of the night is essentially (almost 60%) due to episodes longer than 60 min. Under the T2 or T7 cycles, episodes of short (<10 min) or mid-durations (10–60 min) are not significantly influenced, but the very long episodes of more than 60 min are dramatically less than under T24. These results confirm the fragmentation of wake episodes due to light exposures during subjective night of the T2 and T7 cycles.

Contrary to wake, NREM and REM sleep episodes’ mean duration is not clearly affected by light exposures during subjective night (Figure 2I,L; Supplementary Table S3) in spite of a slight increase in the one of NREM sleep under T2 cycle (Figure 2I). However, the light-induced NREM and REM sleep promotion (Figure 2G,J) relies on a significant increase in the number of episodes of both sleep stages (Figure 2H,K; Supplementary Table S3). Dark exposure during subjective day only moderately decreases the total amount of NREM and REM sleep (mainly due to the effects of CT1 and CT11 pulses) and their number of episodes under both the T2 and T7 cycles, without affecting their mean duration (Supplementary Figure S2D–I; Supplementary Table S4).

### 2.3. The Kinetics of Light and Dark Effects on the Amount and Quality of Sleep and Waking Depends on Light/Dark Pulses Duration

**Figure 3:**
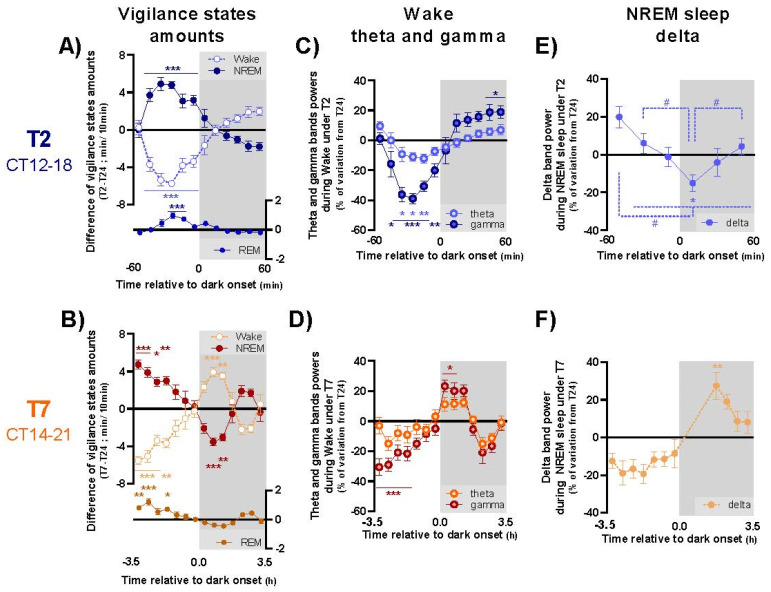
Time course of variation of sleep and waking amount and quality across light and dark exposures under T2 and T7 cycles compared to T24 cycle. Difference of wake (pale blue dotted lines), NREM (dark blue solid lines) and REM (pale blue solid lines) sleep amounts between T2 and T24 cycles per 10 min bins across light and dark exposures averaged for CT12-18 (**A**). Difference of wake (pale orange dotted lines), NREM (dark orange solid lines) and REM (pale orange solid lines) sleep amounts between T7 and T24 cycles per 30 min bins across light and dark exposures for CT14-21 (**B**). Difference of theta (pale blue lines) and gamma (dark blue lines) power during wake between T2 and T24 cycles per 10 min bins across light and dark exposures averaged for CT12-18 (**C**). Difference of theta (pale orange lines) and gamma (dark orange lines) power during wake between T7 and T24 cycles per 30 min bins across light and dark exposures for CT14-21 (**D**). Difference of delta power during NREM sleep between T2 and T24 cycles per 20 min bins across light and dark exposures averaged for CT12-18 (**E**). Difference of delta power during NREM sleep between T7 and T24 cycles per 30 min bins across light and dark exposures for CT14-21 (**F**). Circles represent means ± SEM. Grey areas delineate dark exposures. *, *p* < 0.05, **, *p* < 0.01, ***, *p* < 0.001: difference between results obtained under T2/T7 and T24 cycle for specific time; #, *p* < 0.05 at least: difference between time points for the percentage of variation of delta activity. T24: *n* = 10; T2: *n* = 9; T7: *n* = 7.

To analyze the kinetics of light and dark effects on sleep and waking across 1 h and 3.5 h pulses, the amount of each vigilance state was calculated per 10 min (T2 cycle, Figure 3A) or 30 min bins (T7 cycle; Figure 3B). Similarly, to analyze the influence of 1 h and 3.5 h light and dark pulses on the quality of waking, we performed the same calculation per 10 min (T2) or 30 min (T7) bins for the ECoG theta (4–12 Hz) and gamma (30–70 Hz) activities during wake, two correlates of exploratory behavior and alertness in rodent (Figure 3C,D). During NREM sleep, ECoG delta activity (0.75–4 Hz), a marker of sleep depth, was calculated per 20 min (T2) or 30 min (T7) bins (Figure 3E,F).

Under T2 cycle, light readily induces both NREM and REM sleep, at the cost of wakefulness, for the whole hour of the pulse (Figure 3A; Supplementary Table S5), and this effect is dependent on time of day, as it is of much larger amplitude during early subjective night than during subjective day or late subjective night (Supplementary Figure S3A,B; Supplementary Table S6). Quantitative analysis of the ECoG revealed that light alters the quality of alertness as theta and gamma activities are both decreased during wake (Figure 3C; Supplementary Table S7), an effect that is, again, much greater during the early subjective night (Supplementary Figure S3D,E; Supplementary Table S8). Concerning NREM sleep, a significant decrease in delta-band activity is observed at the beginning of dark pulse under T2 compared to corresponding time points under T24 cycle (Figure 3E; Supplementary Table S9). Moreover, we observe an ultradian evolution across light and dark exposures: declining during light pulses, delta-band power progressively increases during subsequent dark exposure (ANOVA, interaction between light condition and time: F_(2,16)_ = 13.41; *p* < 0.001; Tuckey: *p* < 0.05 at least between the first 20 min of LP and every other time points, and between the first 20 min of DP and 20–40 min of LP and 40–60 min of DP). In comparison to light, dark pulses induce opposite effects, i.e., induce wake at the expense of NREM and REM sleep (Figure 3A, Supplementary Table S5) and promote alertness as shown by the dark-induced promotion of ECoG gamma activity (Figure 3C, Supplementary Table S7). Contrarily to light, dark effects are not as much dependent on time of day, being also present during subjective day (Supplementary Figure S3A,D).

The time-course analysis of the T7 cycle allows us to evaluate the effects of light and dark pulses of longer duration. Under the T7 cycle, the same photic effects on sleep and waking amounts are observed for the first 1 h interval of the 3.5 h light pulses, with similar interaction with the time of day (Figure 3B, Supplementary Figure S3C; Supplementary Tables S5 and S6). Interestingly, when we examine the second period of the pulse, i.e., from 1 h to 3.5 h, we observe an attenuation of light effects on sleep over time, during both subjective night and subjective day. These modifications over time are even greater across dark pulses, especially during subjective night. Indeed, while dark promotes alertness for the first hour of the pulse, with an increase in wake total amounts (Figure 3B, Supplementary Table S5) and of gamma-band activity during wake (Figure 3D; Supplementary Table S7), it inversely decreases alertness and promotes deeper sleep during the second time period of the 3.5 h pulses (Figure 3F; Supplementary Table S9). These effects appear especially during subjective night, compared to subjective day (Supplementary Figure S3F,I, Supplementary Tables S8 and S10). In sum, as a consequence to the kinetics of light/dark effects with time, these data show that ultradian cycles of different durations exert different effects on 24 h sleep and wakefulness distribution and on their quality.

## 3. Discussion

Our study presents major findings concerning the direct effects of alternating light and dark exposures on sleep and waking. We showed that light has major influence on both organization and quality of sleep and wakefulness, which appears especially during early subjective night, with a clear interaction between the direct photic regulation and the circadian regulation. Moreover, the time-course of the amounts and quality of NREM sleep and waking clearly depends on pulses duration, suggesting an interaction of the photic regulation of sleep with the sleep homeostatic process.

The acute sleep-promoting effect of light and wake-promoting effect of dark have already been demonstrated in mice and rats both when applied as a single pulse [5,9,13,15] or in short ultradian LD cycles ([5,14,16,17] but see [18,19]). As suggested in some of these previous studies [13,14,16], our results obtained under the T2 cycle confirm that the most important effect of LD alternation is observed during early subjective night. Importantly, exposure to longer pulses under the T7 cycle (3.5 h vs. 1 h pulse for the T2 cycle) shows the same circadian evolution of light effects, suggesting a strong interaction between direct effects of light and circadian regulation of sleep and waking, independent of the duration of light and dark pulses. This circadian gating of the direct effects of light suggests that it facilitates the photic influence during subjective night, a period during which nocturnal animals spend more time awake, interacting with their environment, than during subjective day. Additionally, the different pathways of photoreception and phototransduction seem to have time of day-dependent sensitivity. Indeed, melanopsin, the photopigment present in intrinsic photosensitive retinal ganglion cells, seems to be particularly involved in light direct effects observed during the early subjective night [5,6,9], which could also explain the circadian modulation of light direct influence.

Contrary to the large light-induced NREM sleep promotion, REM sleep is affected to a far lesser extent by light–dark alternation under short-duration ultradian LD cycle. Given that REM sleep appears only after a consistent period of NREM sleep, short light pulses could be sufficient to promote NREM sleep but longer exposures might be necessary to significantly promote REM sleep. This hypothesis is supported by our results obtained under the T7 cycle, where REM sleep is significantly increased during light pulses compared to dark exposures. These data suggest that REM sleep induction by light might be secondary to the NREM sleep induction.

Even if repeated light and dark pulses modulate sleep and waking amounts in an ultradian way, a circadian variation of sleep–wake distribution remains when mice are exposed to ultradian cycles during 24 h. These results confirm previous observations [5,14,15,19] and suggest that such an exposure limited to 24 h allows circadian clock-related mechanisms to remain expressed, even if circadian photoentrainment is masked by the ultradian LD alternation. On the contrary, more prolonged exposures can lead to a total disappearance of circadian rhythmicity [17,18,20].

In order to go further, dissecting the effects of the T2 and T7 cycles on sleep and waking organization, we evaluated the variation from T24 of each vigilance state amount, their episodes’ number and mean duration, averaged for light and dark exposures, respectively. The time course of these variations was also evaluated across light and dark exposures, along with the ECoG spectral composition of wake and NREM sleep, as a reflection of their quality. The effect of LD alternation is distinct according to vigilance state.

Under short ultradian T2 cycle, inappropriate exposure to light during subjective night induces a dramatic fragmentation of waking, with a clear decrease in the mean duration of episodes, as shown earlier [9], in spite of an increase of almost 100% of their number. Episodes longer than 1 h are particularly affected and split into shorter ones. This fragmentation is accompanied by a decrease in alertness, characterized by a decline of theta and gamma band activities during the whole hour of light exposure. Opposite, but weaker, effects appear when mice are exposed to dark during subjective day, particularly due to the effects of first and last exposures to darkness. These results confirm our previous ones showing a rapid and prolonged increase in theta and gamma activities during wake when mice are exposed to dark in the beginning of the day [5]. In addition, this study is, to our knowledge, the first one to demonstrate that inappropriate light exposure during subjective night not only promotes sleep, at the expense of wakefulness, but also drastically alters alertness, an effect that could reflect a direct effect of light or an indirect consequence of the very significant fragmentation of wake episodes. Contrary to waking, NREM and REM sleep episodes’ mean duration is not dramatically modified under the T2 cycle, in spite of a slight increase in the one of NREM sleep, but the light-induced increase in their total amount relies mostly on an increase in the number of their episodes, which is consistent with a previous report [13]. The time course of NREM sleep amounts modulation by light exposure is consistent with a primary alerting effect of light, with a 10 min delay before sleep induction [10,17]. These modifications of sleep episodes organization are accompanied by a decline of delta power during light exposure and a subsequent increase during dark exposure of subjective night, as a perfect reflection of NREM sleep amounts modulation at the same time points. Therefore, these results suggest an ultradian variation of sleep depth under LD alternation.

Longer light pulses under the T7 cycle induce similar modifications of sleep and waking organization, with an important fragmentation of waking episodes whereas NREM and REM sleep episodes are of the same duration but much more numerous. In the same way as under the T2 cycle, light induces a decrease in alertness, with a reduction in ECoG gamma activity, whereas dark promotes it. Finally, the important decrease in NREM sleep amounts observed during the beginning of dark pulses during subjective night induces a significant rebound of delta activity, in accordance with data published by Szalontai et al. [17], who showed that wake-inducing effect of darkness temporarily blocks the dissipation of the homeostatic sleep drive induced by 6 h of sleep deprivation. Importantly, even if light and dark exposures under the T7 cycle show similar global effects on sleep and waking than under the T2 cycle, the time course of these modulations across light and dark exposures is completely different according to the pulses’ duration. Indeed, while these modulations are maintained along 1 h pulses of light and dark under both the T2 and T7 cycles, light-induced effects are progressively attenuated after one hour of exposure under the T7 cycles, and, more importantly, dark-induced modifications are completely reversed for the second part of the pulse. This time course explains that changes in vigilance states amounts during light and dark pulses in comparison with baseline T24 is of similar level for the T2 and T7 cycles, in spite of the difference of pulses duration. Therefore, when deciphering the acute photic effects under a 1 h of light/1 h of dark schedule, we can assume that within the time frame of 1 h there is not enough time for the animal to build up sleep homeostatic pressure importantly. On the contrary, the duration of 3.5 h pulses is long enough for the dynamics of the homeostatic process to accumulate sleep pressure. Finally, these data suggest that the homeostatic process gets the upper hand over the direct effects of light under ultradian LD cycle with longer light and dark pulses.

Altogether, results obtained under T2 and T7 ultradian LD cycles show that the influence of light and dark on sleep and waking is highly dependent on circadian time and on the duration of pulses. Then, inappropriate lighting exposure according to circadian timing, i.e., light during subjective night or darkness during subjective day, not only modify sleep and wake distribution, but also has a dramatic effect on alertness and sleep depth. Such alterations of vigilance and quality of sleep can have multiple consequences on daily life, among them attention problems, cognitive deficits, car accidents, etc., and these effects are observed even in absence of sleep deprivation. Finally, homeostatic process could, when ultradian light and dark exposures are longer, overtake direct effects of light in the regulation of sleep and waking organization and quality. These data have immediate applications in human, in order to adapt lighting for shift-workers and more generally to optimize societal lighting.

## 4. Materials and Methods

Experimental protocols and animal care were in compliance with European Community Council Directive 2010/63/EU and the current project was approved by the Regional Ethical Committee of Strasbourg for Animal Experimentation and the French Ministry of Higher Education and Research (approval no. #8426-20161129164229760 v6, 19 February 2019).

### 4.1. Subjects and Housing Conditions

C57BL/6 adult male mice were obtained from our animal facility, the Chronobiotron (CNRS UMS 3415). They were housed in transparent Makrolon cages (33 cm × 17 cm × 14 cm) enriched with nesting material, in groups of 2 to 3, in a room with controlled temperature (24 ± 1 °C) and humidity (40 ± 5%) under a 12 h–12 h white polychromatic LD cycle (light on at 7:00 a.m.; 5000 K; 150–200 lux). They had ad libitum access to food and water.

### 4.2. Surgery

At the age of 9 weeks (8.94 ± 1.3), mice were subjected to surgery protocol for electrodes implantation. Under deep anesthesia with intraperitoneal injection of zoletil (40 mg/kg) and xylazine (10 mg/kg), animals were implanted with three gold-plated screws allowing ECoG frontal-parietal derivation and one ground electrode. Two EMG electrodes were inserted into the neck muscles along the back of the skull. All electrodes were then soldered to a connector and cemented to the skull before the skin was sutured. Animals were isolated from the day of surgery until the end of experiments. In total, 13 to 14 days were given to recover from surgery and to habituate to the baseline conditions before any recording began.

### 4.3. Sleep Recordings

ECoG and EMG signals were recorded during (1) 24 h of undisturbed baseline under 12 h–12 h LD cycle called “T24 cycle” (*n* = 10), (2) 24 h of ultradian 1 h light/1 h dark cycle called “T2 cycle” (*n* = 9) and then (3) 24 h of ultradian 3.5 h light/3.5 h dark cycle called “T7 cycle” (*n* = 7). A 12-day period between the two ultradian protocols allowed mice to entirely recover from sleep alterations. Recordings were supported by commercial Compumedics hardware (Neuvo 64–512 Channel EEG HD-LTM, Charlotte, NC, USA) and software (Profusion PSG4 Software, Abbotsford, Australia). ECoG and EMG signals were amplified, filtered, and analog-to-digital converted at 500 Hz for ECoG and 250 Hz for EMG. Sleep stages were manually classified in 4 s epochs according to criteria classically used in mice [20]: non-rapid eye movement (NREM) sleep was distinguished by high-amplitude ECoG dominated by synchronized delta activity (0.75–4 Hz) and a low EMG signal; REM sleep was defined by a regular low-amplitude theta rhythm (4–12 Hz) and a low EMG signal; wake was characterized by a higher and variable EMG and a low-amplitude ECoG with both slower and faster components.

### 4.4. Quantitative Analyses

The amounts of each vigilance state were measured across time within T24 (3 h bins), T2 (1 h bins) and T7 (3.5 h bins) cycles. In order to directly compare ultradian T2 and T7 cycles to T24 one, we calculated the difference between each vigilance state amount during each light and dark pulse of T2 and T7 and corresponding time periods of T24 cycle.

In order to better understand the effects of T2 and T7 cycles on sleep and waking organization, we calculated total amounts, number of episodes and episodes mean duration of wake, NREM and REM sleep under each light and dark exposures. Values were averaged for light vs. dark pulses during subjective day, early (CT12-18) and late (CT17-24) subjective night under T2 or subjective night (CT14-21) under T7, and were represented as percentages of variation of the ones obtained during corresponding time points of T24 cycle [(data under T2 or T7/data under T24)*100-100]. A time-course analysis of sleep and wake distribution variation across light and dark pulses was also performed: the difference of each vigilance state amounts between ultradian LD cycles and T24 was calculated per 10 min (T2 cycle) or 30 min (T7 cycle) bins and averaged for light and dark pulses.

### 4.5. Spectral Analyses

Before processing, the data were cleaned by removing artefacts (values exceeding 3 SD from the mean, together with a 1 s window around selected artefacts). ECoG power spectra were calculated using Chronux signal processing toolbox [21] with a time-frequency product of three and five tapers. Fast Fourier transform was performed on a moving window (window 4 s; overlap 1 s) and power of delta (0.75–4 Hz), theta (4–12 Hz) and gamma (30–70 Hz) frequency bands was analyzed.

The time course of theta and gamma band power during wake episodes was averaged per bins of 10—(T2) or 30—(T7) min and represented as percentages of variation from the one under T24 at corresponding time points (per bins of 1 h for comparison to T2 or 3.5 h for comparison to T7 cycle). Delta band activity was also measured per 20—(T2) or 30—(T7) min bins during NREM sleep episodes. These data were averaged for subjective day, early (CT12-18) and late (CT17-24) subjective night under T2 or subjective night (CT14-21) under T7.

### 4.6. Statistical Analysis

Statistical analysis was realized using Statistica (Statsoft, Tulsa, OK, USA; version 13). Repeated-measures analysis of variance (ANOVA) were used to measure time-course changes in vigilance states amounts and in frequency bands power according to cycle and light condition. The effect of cycle was evaluated on the total amounts, episodes number and mean duration of each vigilance state. HSD Tukey multiple range tests were then performed to determine post hoc significance. The threshold for rejecting the null hypothesis was 0.05 throughout.

## Data Availability

Not applicable.

## Supplementary Materials

The following supporting information can be downloaded at: https://www.mdpi.com/article/10.3390/clockssleep4020019/s1, Figure S1: Wake amounts distribution according to episodes duration under T24, T2 and T7 cycles; Figure S2: Influence of light and dark pulses on wake, NREM and REM sleep organization under T2 and T7, compared to T24 cycle; Figure S3: Time-course of variation of sleep and waking amount and quality across light and dark exposures under the T2 and T7 cycles compared to T24 cycle; Table S1: Statistical results corresponding to Figure 1B,C; Table S2: Statistical results corresponding to Figure 1D,E; Table S3: Statistical results corresponding to Figure 2; Table S4: Statistical results corresponding to Supplementary Figure S2; Table S5: Statistical results corresponding to Figure 3A,B; Table S6: Statistical results corresponding to Supplementary Figure S3A–C; Table S7: Statistical results corresponding to Figure 3C,D; Table S8: Statistical results corresponding to Supplementary Figure S3D–F; Table S9: Statistical results corresponding to Figure 3E,F; Table S10: Statistical results corresponding to Supplementary Figure S3G–I.
