# Supplementary material for: Direct Effects of Light on Sleep under Ultradian Light-Dark Cycles Depend on Circadian Time and Pulses Duration
Source: Clocks Sleep. 2022 Mar 24;4(2):208–18. doi: 10.3390/clockssleep4020019 (PMC9036312; doi:10.3390/clockssleep4020019)
Supplement: Supplementary file 1 [file clockssleep-04-00019-s001.zip › clockssleep-1586471 Supplementary materials.pdf]

Supplementary materials:

To specify the distribution of wake episodes, and because long episodes contributed substantially to the time spent in wake during the night, time-weighted frequency histograms were computed. To do this, all episodes were separated into four bins of exponentially increasing duration (<10, 10-30, 30-60, >60 min). The amount of wake in each bin was normalized by the total amount of wake in each animal during (subjective) day and night and then averaged for the whole group.

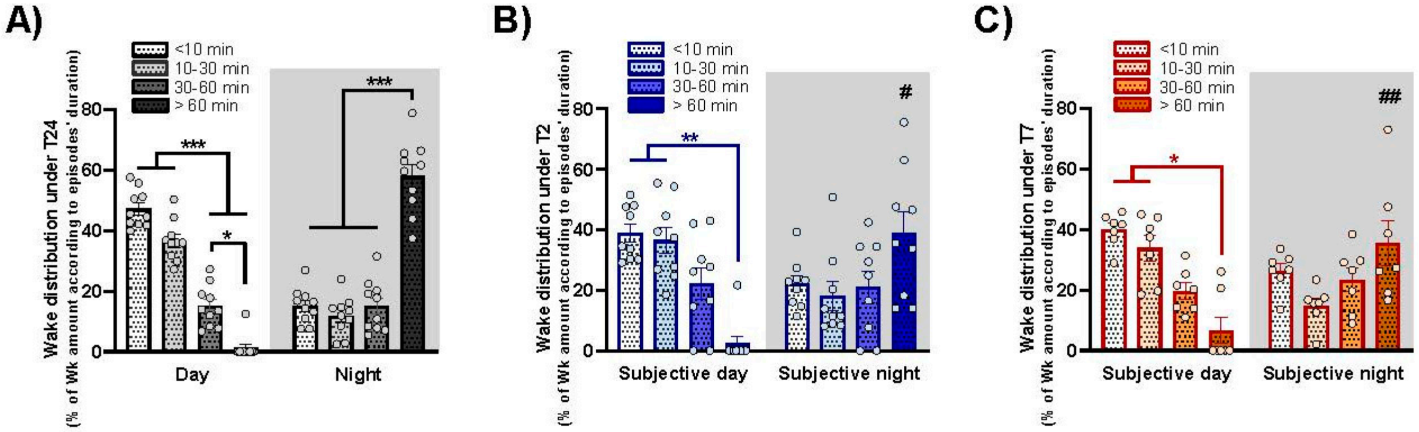

Supplementary Figure S1: Wake amounts distribution according to episodes duration under T24, T2 and T7 cycles. Percentage of wake total duration in function of wake episodes' duration, during day and night under T24 (**A**), and during subjective day and night under T2 (**B**) and T7 (**C**) cycles. Histograms represent means ± SEM, circles represent individual values. Dark grey areas delineate dark exposures under T24 cycle; pale grey areas delineate subjective dark exposures under T2 or T7 cycles. *, $p < 0.05$, **, $p < 0.01$, ***, $p < 0.001$: difference between episodes' duration during the same phase; #, $p < 0.05$, ##, $p < 0.01$: difference from the same duration under T24 cycle.

Supplementary Results S1:

Under T24 cycle, the distribution of wake amounts according to episodes' duration is totally different between day and night (Supplementary Figure S1A). During the day, episodes lasting less than 10 min or lasting 10 to 30 min almost contribute to 85% of total wake amounts, being significantly higher than longer episodes lasting more than 30 min (ANOVA, interaction between the effect of phase and episodes duration: $F_{(3,27)} = 129.45$, $p < 0.001$; Tukey: $p < 0.001$ between bins < 30 min and > 30 min). Moreover, the part of wake amount due to episodes longer than 60 min is almost nothing (Tukey: $p < 0.05$ between bins 30-60 min and > 60 min). On the contrary, total wake amounts of the night is essentially due to very long episodes of more than 60 min (almost 60%) whereas shorter episodes are less represented (Tukey: $p < 0.001$ between bins > 60 min and every other bins). Under T2 (Supplementary Figure S1B) and T7 cycles (Supplementary Figure S1C), distribution of wake is quite similar to the one of T24 during subjective day, with a significant difference between the contribution of episodes shorter than 30 min and the one of episodes longer than 60 min (ANOVA, interaction between the effect of phase and episodes duration: T2: $F_{(3,24)} = 10.48$, $p < 0.001$; Tukey: $p < 0.01$ between bins < 30 min and > 60 min; T7: $F_{(3,18)} = 9.23$, $p < 0.001$; Tukey: $p < 0.05$ between bins < 30 min and > 60 min). However, during subjective night, episodes longer than 60 min contribute less to total wake amounts than under T24 cycle (ANOVA, interaction between the effect of cycle, phase and episodes duration: T2: $F_{(3,24)} = 4.37$, $p < 0.05$; T7: $F_{(3,18)} = 11.30$, $p < 0.001$; for both, Tukey: $p < 0.05$ between bins > 60 min during subjective night of T2 compared to the one of the night of T24).

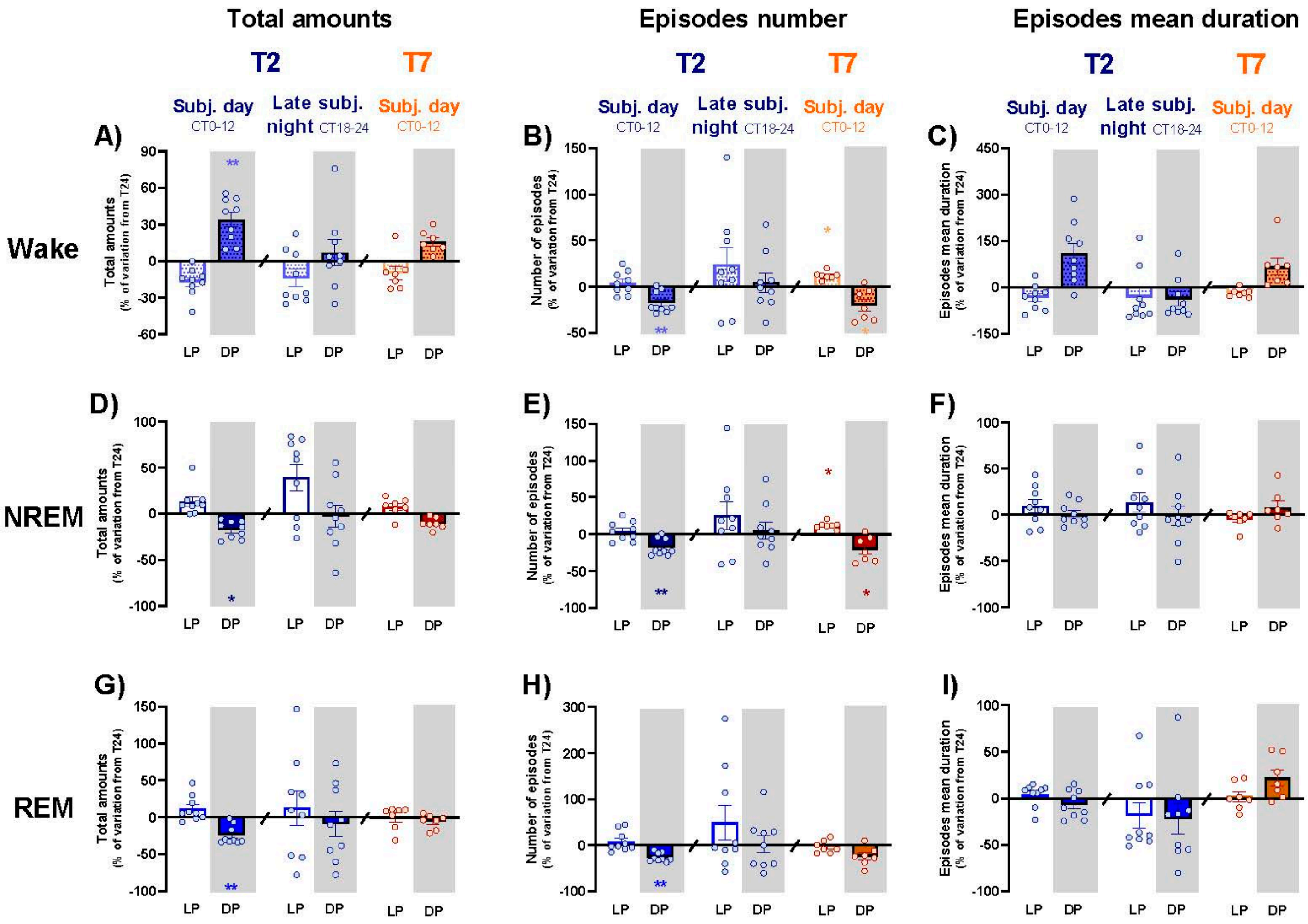

Supplementary Figure S2: Influence of light and dark pulses on wake, NREM and REM sleep organization under T2 and T7, compared to T24 cycle. The percentage of variation from T24 cycle for total amounts of wake (**A**), NREM (**D**) and REM (**G**) sleep, number of episodes of wake (**B**), NREM (**E**) and REM (**H**) sleep, and for episodes mean duration of wake (**C**), NREM (**F**) and REM (**I**) sleep during light (empty bars) and dark (filled bars) pulses averaged for subjective day (CT0-12) under T2 (blue, left) or T7 (orange, right) cycles, and during late subjective night (CT18-24; blue, middle) of T2 cycle. Histograms represent means ± SEM, circles represent individual values. Grey areas delineate dark exposures. *, $p < 0.05$, **, $p < 0.01$: difference between results obtained under T2/T7 and T24 cycle. LP: light pulse; DP: dark pulse. T24: n = 10; T2: n = 9; T7: n = 7.

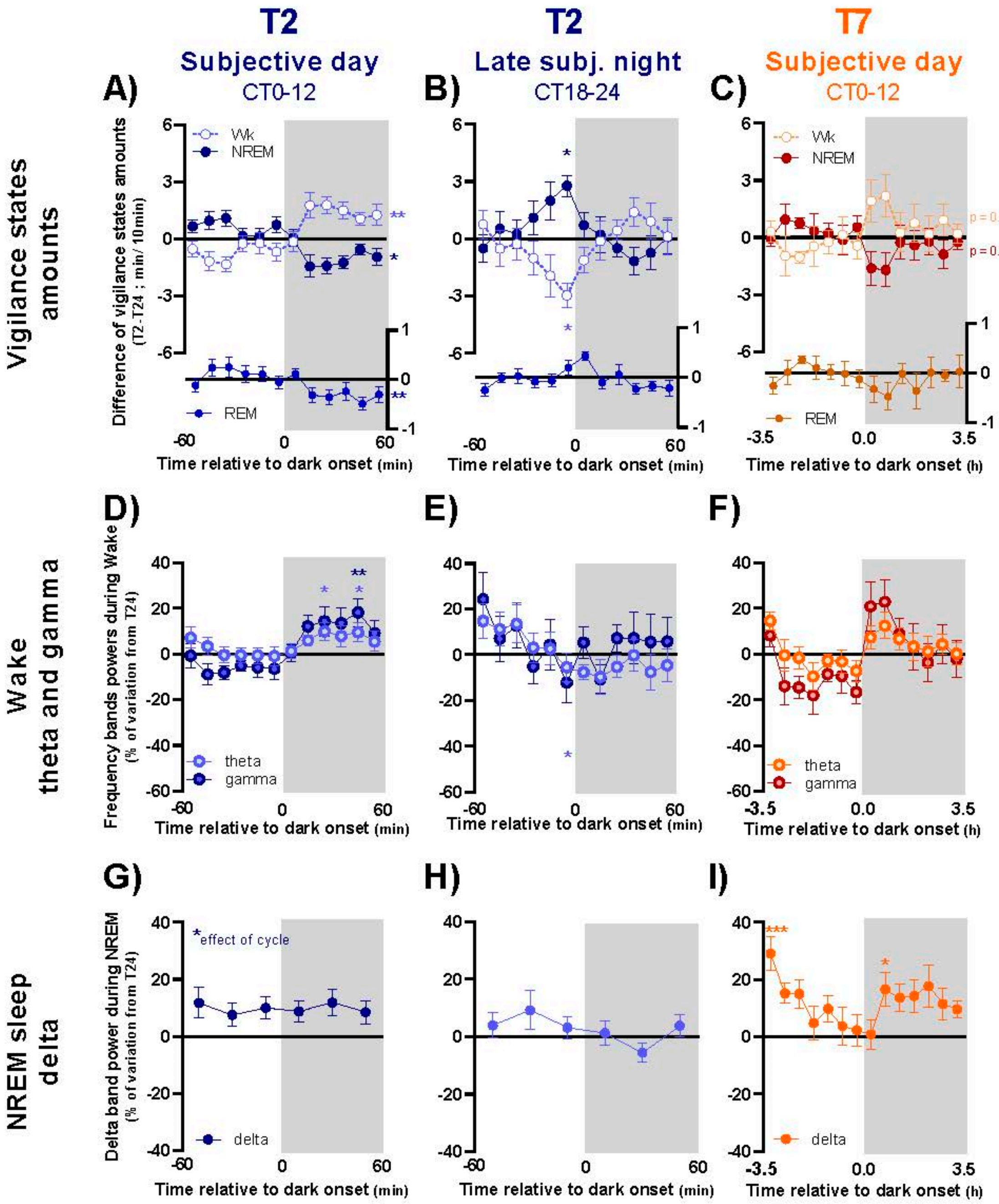

Supplementary Figure S3. Time-course of variation of sleep and waking amount and quality across light and dark exposures under T2 and T7 cycles compared to T24 cycle. Difference of wake (pale blue dotted lines), NREM (dark blue solid lines) and REM (pale blue solid lines) sleep amounts between T2 and T24 cycles per 10 min bins across light and dark exposures averaged for subjective day (CT0-12, **A**) or late subjective night (CT18-24, **B**). Difference of wake (pale orange dotted lines), NREM (dark orange solid lines) and REM (pale orange solid lines) sleep amounts between T7 and T24 cycles per 30 min bins across light and dark exposures for subjective day (CT0-12, **C**). Difference of theta (pale blue lines) and gamma (dark blue lines) power during wake between T2 and T24 cycles per 10 min bins across light and dark exposures averaged for subjective day (CT0-12, **D**) or late subjective night (CT18-24, **E**). Difference of theta (pale orange lines) and gamma (dark orange lines) power during wake between T7 and T24 cycles per 30 min bins across light and dark exposures for subjective day (CT0-12, **F**). Difference of delta power during NREM sleep between T2 and T24 cycles per 20 min bins across light and dark exposures averaged for subjective day (CT0-12, **G**) or late subjective night (CT18-24,

H). Difference of delta power during NREM sleep between T7 and T24 cycles per 30 min bins across light and dark exposures for subjective day (CT0-12, **I**). Circles represent means ± SEM. Grey areas delineate dark exposures. *, $p < 0.05$, **, $p < 0.01$, ***, $p < 0.001$: difference between results obtained under T2/T7 and T24 cycle for specific time points (above circles; interaction cycle x light condition x time), for the entire DP (on the right of the figure; interaction cycle x light condition) or for the entire period (effect of cycle). T24: n = 10; T2: n = 9; T7: n = 7.

Time \ Variable		Wake amounts	NREM amounts	REM amounts
T2		$F_{(23,184)} = 13.49$, $p < 0.001$	$F_{(23,184)} = 13.02$, $p < 0.001$	$F_{(23,184)} = 11.30$, $p < 0.001$
	CT0-1 vs CT1-2	p = 0.80	p = 0.54	p = 1.00
	CT2-3 vs CT3-4	p = 0.35	p = 0.56	p = 0.19
	CT4-5 vs CT5-6	p = 1.00	p = 1.00	p = 1.00
	CT6-7 vs CT7-8	p = 0.97	p = 0.99	p = 0.98
	CT8-9 vs CT9-10	p = 1.00	p = 1.00	p = 1.00
	CT10-11 vs CT11-12	p < 0.05	p < 0.05	p = 0.32
	CT12-13 vs CT13-14	p < 0.001	p < 0.001	p = 0.24
	CT14-15 vs CT15-16	p < 0.001	p < 0.001	p = 0.98
	CT16-17 vs CT17-18	p < 0.001	p < 0.001	p = 0.86
	CT18-19 vs CT19-20	p = 1.00	p = 1.00	p = 1.00
	CT20-21vs CT21-22	p = 0.31	p = 0.16	p = 1.00
	CT22-23 vs CT23-24	p = 1.00	p = 1.00	p = 1.00
T7		$F_{(6,36)} = 20.01$, $p < 0.001$	$F_{(6,36)} = 16.52$, $p < 0.001$	$F_{(6,36)} = 26.80$, $p < 0.001$
	CT0-3.5 vs CT3.5-7	p =1.00	p = 0.99	p = 0.96
	CT7-10.5 vs CT10.5-14	p < 0.001	p < 0.001	p < 0.001
	CT14-17.5 vs CT17.5-21	p < 0.001	p < 0.001	p < 0.01

Supplementary Table S1: Statistical results corresponding to Figure 1B-C. Comparison of wake, NREM and REM sleep amounts between consecutive light and dark pulses under T2 and T7 cycles: results of ANOVAs (effect of time) and HSD Tuckey's tests. Significant differences are in black whereas non-significant results are in grey.

Time x light condition \ Variable		Wake amounts	NREM amounts	REM amounts
T2		$F_{(23,184)} = 7.54$, $p < 0.001$	$F_{(23,184)} = 7.83$, $p < 0.001$	$F_{(23,184)} = 3.84$, $p < 0.001$
	CT0-1	p = 1.00	p = 1.00	p = 1.00
	CT1-2	p = 0.13	p = 0.26	p = 0.13
	CT2-3	p = 0.96	p = 0.99	p = 0.85
	CT3-4	p = 1.00	p = 1.00	p = 0.92
	CT4-5	p = 1.00	p = 1.00	p = 0.67
	CT5-6	p = 1.00	p = 1.00	p = 1.00
	CT6-7	p = 1.00	p = 1.00	p = 1.00
	CT7-8	p = 1.00	p = 1.00	p = 1.00
	CT8-9	p = 1.00	p = 1.00	p = 1.00
	CT9-10	p = 1.00	p = 1.00	p = 1.00
	CT10-11	p = 1.00	p = 1.00	p = 1.00
	CT11-12	p = 0.07	p = 0.10	p = 0.45
	CT12-13	p < 0.001	p < 0.001	p = 0.052
	CT13-14	p = 1.00	p = 1.00	p = 1.00
	CT14-15	p < 0.001	p < 0.001	p = 0.83
	CT15-16	p = 1.00	p = 1.00	p = 1.00
	CT16-17	p < 0.05	p < 0.01	p = 1.00
	CT17-18	p = 0.87	p = 0.75	p = 1.00
	CT18-19	p = 1.00	p = 1.00	p = 1.00
	CT19-20	p = 1.00	p = 1.00	p = 1.00
	CT20-21	p = 0.99	p = 0.89	p = 1.00
	CT21-22	p = 1.00	p = 1.00	p = 1.00
	CT22-23	p = 1.00	p = 0.99	p = 1.00
	CT23-24	p = 1.00	p = 1.00	p = 1.00
T7		$F_{(6,36)} = 21.72$, $p < 0.001$	$F_{(6,36)} = 20.70$, $p < 0.001$	$F_{(6,36)} = 15.82$ $p < 0.001$
	CT0-3.5	p =1.00	p =1.00	p = 0.33
	CT3.5-7	p =0.21	p =0.19	p = 0.84
	CT7-10.5	p =0,09	p = 0.13	p = 0.18
	CT10.5-14	p =1.00	p =1.00	p = 0.95
	CT14-17.5	p < 0.001	p < 0.001	p < 0.001
	CT17.5-21	p =0,14	p = 0.10	p = 0.97
	CT21-24	p < 0.01	p < 0.01	p = 0.07

Supplementary Table S2: Statistical results corresponding to Figure 1D-E. Comparison of wake, NREM and REM sleep amounts between each light and dark exposure under T2 and T7 cycles and corresponding time points under T24 cycle: results of ANOVAs (interaction between time and light condition) and HSD Tuckey's tests. Significant differences are in black whereas non-significant results are in grey. Grey compartments represent dark pulses.

Variable	Cycle x light condition	T2 (CT12-18) LP	T2 (CT12-18) DP	T7 (CT14-21) LP	T7 (CT14-21) DP
Wk	Total amount	$F_{(1,8)} = 82.19$, $p < 0.001$		$F_{(1,6)} = 88.92$, $p < 0.001$	
		$p < 0.001$	$p = 0.26$	$p < 0.001$	$p = 0.07$
	Number of episodes	$F_{(1,8)} = 49.03$, $p < 0.001$		$F_{(1,6)} = 46.92$, $p < 0.001$	
		$p < 0.001$	$p = 0.36$	$p < 0.01$	$p = 0.27$
	Mean duration	$F_{(1,8)} = 14.95$, $p < 0.01$		$F_{(1,6)} = 7.54$, $p < 0.05$	
		$p < 0.01$	$p = 0.86$	$p = 0.07$	$p = 0.90$
NREM	Total amount	$F_{(1,8)} = 94.13$, $p < 0.001$		$F_{(1,6)} = 73.87$, $p < 0.001$	
		$p < 0.001$	$p = 0.14$	$p < 0.001$	$p = 0.07$
	Number of episodes	$F_{(1,8)} = 48.95$, $p < 0.001$		$F_{(1,6)} = 47.69$, $p < 0.001$	
		$p < 0.001$	$p = 0.38$	$p < 0.01$	$p = 0.25$
	Mean duration	$F_{(1,8)} = 29.66$, $p < 0.001$		$F_{(1,6)} = 0.85$, $p = 0.39$	
		$p < 0.01$	$p = 0.054$		
REM	Total amount	$F_{(1,8)} = 23.42$, $p < 0.01$		$F_{(1,6)} = 38.21$, $p < 0.001$	
		$p < 0.001$	$p = 1.00$	$p < 0.01$	$p = 0.58$
	Number of episodes	$F_{(1,8)} = 27.18$, $p < 0.001$		$F_{(1,6)} = 59.44$, $p < 0.001$	
		$p < 0.01$	$p = 0.80$	$p < 0.001$	$p = 0.26$
	Mean duration	$F_{(1,8)} = 0.08$, $p = 0.79$		$F_{(1,6)} = 1.81$, $p = 0.23$	

Supplementary Table S3: Statistical results corresponding to Figure 2. Comparison of wake, NREM and REM sleep total amounts, number of episodes and episodes' mean duration averaged for light and dark exposures during subjective night under T2 (CT12-18) and T7 (CT14-21) cycles to the one obtained during corresponding time points under T24 cycle: results of ANOVAs (interaction between cycle and light condition) and HSD Tuckey's tests. Significant differences are in black whereas non-significant results are in grey.

<table>
<tr><th colspan="2" rowspan="3">Cycle x light condition
Variable</th><th colspan="4">T2</th><th colspan="2">T7</th></tr>
<tr><th colspan="2">Subj. day CT0-12</th><th colspan="2">Late subj. Night CT18-24</th><th colspan="2">Subjective day CT0-12</th></tr>
<tr><th>LP</th><th>DP</th><th>LP</th><th>DP</th><th>LP</th><th>DP</th></tr>
<tr><td rowspan="6">Wk</td><td rowspan="2">Total amount</td><td colspan="2">$F_{(1,8)} = 30.61, p < 0.001$</td><td colspan="2" rowspan="2">$F_{(1,8)} = 2.10, p = 0.19$</td><td colspan="2">$F_{(1,6)} = 10.34, p < 0.05$</td></tr>
<tr><td>$p = 0.08$</td><td>$p < 0.01$</td><td>$p = 0.15$</td><td>$p = 0.29$</td></tr>
<tr><td rowspan="2">Number of episodes</td><td colspan="2">$F_{(1,8)} = 17.23, p < 0.01$</td><td colspan="2" rowspan="2">$F_{(1,8)} = 1.06, p =0.33$</td><td colspan="2">$F_{(1,6)} = 45.19, p < 0.001$</td></tr>
<tr><td>$p = 0.76$</td><td>$p < 0.01$</td><td>$p < 0.05$</td><td>$p < 0.05$</td></tr>
<tr><td rowspan="2">Mean duration</td><td colspan="2">$F_{(1,8)} = 6.50, p < 0.05$</td><td colspan="2" rowspan="2">$F_{(1,8)} = 0.01, p =0.91$</td><td colspan="2">$F_{(1,6)} = 17.27, p < 0.01$</td></tr>
<tr><td>$p = 0.22$</td><td>$p = 0.50$</td><td>$p = 0.16$</td><td>$p = 0.05$</td></tr>
<tr><td rowspan="6">NREM</td><td rowspan="2">Total amount</td><td colspan="2">$F_{(1,8)} = 26.43, p < 0.001$</td><td colspan="2">$F_{(1,8)} = 26.43, p < 0.001$</td><td colspan="2">$F_{(1,6)} = 15.87, p < 0.01$</td></tr>
<tr><td>$p = 0.08$</td><td>$p < 0.05$</td><td>$p = 0.40$</td><td>$p = 0.69$</td><td>$p = 0.06$</td><td>$p = 0.19$</td></tr>
<tr><td rowspan="2">Number of episodes</td><td colspan="2">$F_{(1,8)} = 16.78, p < 0.01$</td><td colspan="2" rowspan="2">$F_{(1,8)} = 1.06, p =0.33$</td><td colspan="2">$F_{(1,6)} = 44.90, p < 0.001$</td></tr>
<tr><td>$p = 0.82$</td><td>$p < 0.01$</td><td>$p < 0.05$</td><td>$p < 0.05$</td></tr>
<tr><td rowspan="2">Mean duration</td><td colspan="2" rowspan="2">$F_{(1,8)} = 7.79, p =0.22$</td><td colspan="2" rowspan="2">$F_{(1,8)} = 1.08, p =0.33$</td><td colspan="2">$F_{(1,6)} = 7.44, p < 0.05$</td></tr>
<tr><td>$p = 0.22$</td><td>$p = 0.43$</td></tr>
<tr><td rowspan="6">REM</td><td rowspan="2">Total amount</td><td colspan="2">$F_{(1,8)} = 21.01, p < 0.01$</td><td colspan="2">$F_{(1,8)} = 21.01, p < 0.01$</td><td colspan="2" rowspan="2">$F_{(1,6)} = 0.42, p = 0.54$</td></tr>
<tr><td>$p = 0.41$</td><td>$p < 0.01$</td><td>$p = 0.40$</td><td>$p = 0.69$</td></tr>
<tr><td rowspan="2">Number of episodes</td><td colspan="2">$F_{(1,8)} = 15.02, p < 0.01$</td><td colspan="2" rowspan="2">$F_{(1,8)} = 0.36, p =0.56$</td><td colspan="2" rowspan="2">$F_{(1,6)} = 4.05, p < 0.09$</td></tr>
<tr><td>$p = 0.75$</td><td>$p < 0.01$</td></tr>
<tr><td rowspan="2">Mean duration</td><td colspan="2" rowspan="2">$F_{(1,8)} = 2.05, p =0.19$</td><td colspan="2" rowspan="2">$F_{(1,8)} = 0.00, p =0.98$</td><td colspan="2" rowspan="2">$F_{(1,6)} = 1.45, p < 0.27$</td></tr>
<tr></tr>
</table>

Supplementary Table S4: Statistical results corresponding to Supplementary Figure S2. Comparison of wake, NREM and REM sleep total amounts, number of episodes and episodes' mean duration averaged for light and dark exposures during subjective day (CT0-12) under T2 or T7 cycles, and during late subjective night under T2 cycle (CT18-24) to the one obtained during corresponding time points under T24 cycle: results of ANOVAs (interaction between cycle and light condition) and HSD Tuckey's tests. Significant differences are in black whereas non-significant results are in grey.

Cycle x light condition x time \ Variable			Wake amounts	NREM amounts	REM amounts
T2			$F_{(5,40)} = 18.08$, $p < 0.001$	$F_{(5,40)} = 12.67$, $p < 0.001$	$F_{(5,40)} = 13.58$, $p < 0.001$
	CT12-18	0-10 min	$p = 1.00$	$p = 1.00$	$p = 0.99$
		10-20 min	$p < 0.001$	$p < 0.001$	$p = 1.00$
		20-30 min	$p < 0.001$	$p < 0.001$	$p = 0.08$
		30-40 min	$p < 0.001$	$p < 0.001$	$p < 0.001$
		40-50 min	$p < 0.001$	$p < 0.001$	$p < 0.001$
		50-60 min	$p < 0.001$	$p < 0.001$	$p = 0.98$
		0-10 min	$p = 0.28$	$p = 0.77$	$p = 0.12$
		10-20 min	$p = 1.00$	$p = 1.00$	$p = 1.00$
		20-30 min	$p = 1.00$	$p = 1.00$	$p = 1.00$
		30-40 min	$p = 0.83$	$p = 0.93$	$p = 1.00$
		40-50 min	$p = 0.12$	$p = 0.18$	$p = 1.00$
		50-60 min	$p = 0.09$	$p = 0.16$	$p = 1.00$
T7			$F_{(6,36)} = 18.10$, $p < 0.001$	$F_{(6,36)} = 15.34$, $p < 0.001$	$F_{(6,36)} = 10.24$, $p < 0.001$
	CT14-21	0-30 min	$p < 0.001$	$p < 0.001$	$p < 0.01$
		30-60 min	$p < 0.001$	$p < 0.001$	$p < 0.001$
		60-90 min	$p < 0.001$	$p < 0.05$	$p = 0.17$
		90-120 min	$p < 0.01$	$p < 0.01$	$p < 0.05$
		120-150 min	$p = 0.35$	$p = 0.47$	$p = 0.95$
		150-180 min	$p = 1.00$	$p = 1.00$	$p = 1.00$
		180-210 min	$p = 1.00$	$p = 1.00$	$p = 1.00$
		0-30 min	$p = 0.20$	$p = 0.22$	$p = 1.00$
		30-60 min	$p < 0.001$	$p < 0.001$	$p = 0.64$
		60-90 min	$p < 0.01$	$p < 0.01$	$p = 0.39$
		90-120 min	$p = 1.00$	$p = 1.00$	$p = 1.00$
		120-150 min	$p = 0.28$	$p = 0.39$	$p = 0.92$
		150-180 min	$p = 0.33$	$p = 0.60$	$p = 0.45$
		180-210 min	$p = 1.00$	$p = 1.00$	$p = 1.00$

Supplementary Table S5: Statistical results corresponding to Figure 3A-B. Comparison of wake, NREM and REM sleep total amounts across light and dark pulses, averaged for CT12-18 under T2 (10 min bins) and CT14-21 under T7 (30 min bins) cycles and values obtained during corresponding time points under T24 cycle: results of ANOVAs (interaction between cycle, light condition and time) and HSD Tuckey's tests. Significant differences are in black whereas non-significant results are in grey. Grey compartments represent dark pulses.

Cycle x light condition x time \ Variable			Wake amounts	NREM amounts	REM amounts
T2	cycle x light condition		$F_{(5,40)} = 1.70$, $p = 0.16$<br>$F_{(1,8)} = 30.61$, $p < 0.001$*	$F_{(5,40)} = 1.46$, $p = 0.22$<br>$F_{(1,8)} = 26.43$, $p < 0.001$*	$F_{(5,40)} = 1.82$, $p = 0.13$<br>$F_{(1,8)} = 21.01$, $p < 0.01$*
	**Subj. Day** CT0-12	0-60 min	$p = 0.08$	$p = 0.08$	$p = 0.41$
		0-60 min	$p < 0.01$	$p < 0.05$	$p < 0.01$
			$F_{(5,40)} = 3.31$, $p < 0.05$	$F_{(5,40)} = 2.48$, $p < 0.05$	$F_{(5,40)} = 5.24$, $p < 0.001$
	**Late subj. Night** CT18-24	0-10 min	$p = 1.00$	$p = 1.00$	$p = 0.84$
		10-20 min	$p = 1.00$	$p = 1.00$	$p = 1.00$
		20-30 min	$p = 1.00$	$p = 1.00$	$p = 1.00$
		30-40 min	$p = 1.00$	$p = 0.99$	$p = 1.00$
		40-50 min	$p = 0.59$	$p = 0.45$	$p = 1.00$
		50-60 min	$p < 0.05$	$p < 0.05$	$p = 0.98$
		0-10 min	$p = 1.00$	$p = 1.00$	$p = 0.07$
		10-20 min	$p = 1.00$	$p = 1.00$	$p = 1.00$
		20-30 min	$p = 1.00$	$p = 1.00$	$p = 1.00$
		30-40 min	$p = 0.96$	$p = 0.99$	$p = 0.92$
		40-50 min	$p = 1.00$	$p = 1.00$	$p = 1.00$
		50-60 min	$p = 1.00$	$p = 1.00$	$p = 0.96$
T7	cycle x light condition		$F_{(6,36)} = 0.42$, $p = 0.86$<br>$F_{(1,6)} = 10.87$, $p < 0.05$*	$F_{(6,36)} = 0.48$, $p = 0.82$<br>$F_{(1,6)} = 12.26$, $p < 0.05$*	$F_{(6,36)} = 0.34$, $p = 0.91$<br>$F_{(1,6)} = 3.23$, $p = 0.12$*
	**Subj. Day** CT0-12	0-210 min	$p = 0.48$	$p = 0.38$	
		0-210 min	$p = 0.07$	$p = 0.07$	

<u>Supplementary Table S6:</u> Statistical results corresponding to Supplementary Figure S3A-C. Comparison of Wake, NREM and REM sleep total amounts across light and dark pulses, averaged for subjective day (CT0-12) under T2 (10 min bins) or T7 (30 min bins) cycles, and for late subjective night under T2 cycle (CT18-24) and values obtained during corresponding time points under T24 cycle: results of ANOVAs (interaction between cycle, light condition and time, and, if non-significant, *interaction between cycle and light condition) and HSD Tuckey's tests. Significant differences are in black whereas non-significant results are in grey. Grey compartments represent dark pulses.

Cycle x light condition x time (Variable)			Theta-band power	Gamma-band power
T2	CT12-18		$F_{(5,40)} = 12.30$ $p < 0.001$	$F_{(5,40)} = 17.62$, $p < 0.001$
		0-10 min	p = 0.13	p = 1.00
		10-20 min	p = 1.00	p < 0.05
		20-30 min	p < 0.05	p < 0.001
		30-40 min	p < 0.05	p < 0.001
		40-50 min	p < 0.01	p < 0.001
		50-60 min	p = 0.45	p < 0.01
		0-10 min	p = 0.74	p = 0.61
		10-20 min	p = 1.00	p = 0.78
		20-30 min	p = 1.00	p = 0.43
		30-40 min	p = 0.99	p = 0.07
		40-50 min	p = 0.91	p < 0.05
		50-60 min	p = 0.62	p < 0.05
T7	CT14-21		$F_{(6,36)} = 7.24$, $p < 0.001$	$F_{(6,36)} = 13.92$, $p < 0.001$
		0-30 min	p = 1.00	p < 0.001
		30-60 min	p = 0.06	p < 0.001
		60-90 min	p = 0.76	p < 0.001
		90-120 min	p = 0.85	p < 0.001
		120-150 min	p = 1.00	p = 0.24
		150-180 min	p = 1.00	p = 0.98
		180-210 min	p = 1.00	p = 1.00
		0-30 min	p = 0.29	p < 0.05
		30-60 min	p = 0.28	p < 0.05
		60-90 min	p = 0.22	p = 0.053
		90-120 min	p = 1.00	p = 1.00
		120-150 min	p = 0.11	p = 0.11
		150-180 min	p = 0.56	p = 0.29
		180-210 min	p = 1.00	p = 1.00

Supplementary Table S7: Statistical results corresponding to Figure 3C-D. Comparison of theta and gamma-band power during wake across light and dark pulses, averaged for CT12-18 under T2 (10 min bins) and CT14-21 under T7 (30 min bins) cycles and values obtained during corresponding time points under T24 cycle: results of ANOVAs (interaction between cycle, light condition and time) and HSD Tuckey's tests. Significant differences are in black whereas non-significant results are in grey. Grey compartments represent dark pulses.

Cycle x light condition x time \ Variable			Theta-band power	Gamma-band power
T2	Subj. Day CT0-12		$F_{(5,40)} = 4.62, p < 0.01$	$F_{(5,40)} = 3.35, p < 0.05$
		0-10 min	$p = 0.32$	$p = 1.00$
		10-20 min	$p = 1.00$	$p = 0.08$
		20-30 min	$p = 1.00$	$p = 0.12$
		30-40 min	$p = 1.00$	$p = 0.59$
		40-50 min	$p = 1.00$	$p = 0.24$
		50-60 min	$p = 1.00$	$p = 0.42$
		0-10 min	$p = 1.00$	$p = 1.00$
		10-20 min	$p = 0.82$	$p = 0.37$
		20-30 min	$p < 0.05$	$p = 0.12$
		30-40 min	$p = 0.17$	$p = 0.22$
		40-50 min	$p < 0.05$	$p < 0.01$
		50-60 min	$p = 0.37$	$p = 0.73$
	Late subj. night CT18-24		$F_{(5,40)} = 2.99$ $p < 0.05$	$F_{(5,40)} = 0.64$ $p = 0.67$
		0-10 min	$p = 0.87$	
		10-20 min	$p = 1.00$	
		20-30 min	$p = 1.00$	
		30-40 min	$p = 1.00$	
		40-50 min	$p = 1.00$	
		50-60 min	$p < 0.05$	
		0-10 min	$p = 0.07$	
		10-20 min	$p = 0.21$	
		20-30 min	$p = 1.00$	
		30-40 min	$p = 1.00$	
		40-50 min	$p = 0.59$	
		50-60 min	$p = 0.27$	
T7	cycle x light condition		$F_{(6,36)} = 1.69$ $p = 0.15$<br>$F_{(1,6)} = 6.05, p < 0.05$*	$F_{(6,36)} = 1.07, p = 0.40$
	Subj. Day CT0-12	0-210 min	$p = 0.91$	
		0-210 min	$p = 0.10$	

<u>Supplementary Table S8:</u> Statistical results corresponding to Supplementary Figure S3D-F. Comparison of theta and gamma-band power during Wake across light and dark pulses, averaged for subjective day (CT0-12) under T2 (10-min bins) or T7 (30-min bins) cycles, and for late subjective night under T2 cycle (CT18-24) and values obtained during corresponding time points under T24 cycle: results of ANOVAs (interaction between cycle, light condition and time, and, if non-significant, *interaction between cycle and light condition) and HSD Tuckey's tests. Significant differences are in black whereas non-significant results are in grey. Grey compartments represent dark pulses.

Cycle x light condition x time			Delta-band power
T2	CT12-18		$F_{(2,16)} = 9.04$ $p < 0.001$
		0-20 min	$p = 0.07$
		20-40 min	$p = 1.00$
		40-60 min	$p = 1.00$
		0-20 min	$p < 0.05$
		20-40 min	$p = 0.28$
		40-60 min	$p = 1.00$
T7	cycle x time		$F_{(10,60)} = 9.51$, $p < 0.001$
	CT14-21	0-30 min	$p = 0.73$
		30-60 min	$p = 0.08$
		60-90 min	$p = 0.31$
		90-120 min	$p = 0.10$
		120-150 min	$p = 0.18$
		150-180 min	$p = 0.40$
		180-210 min	$p = 0.57$
		0-90 min	
		90-120 min	$p < 0.01$
		120-150 min	$p = 0.15$
		150-180 min	$p = 0.99$
		180-210 min	$p = 0.99$

Supplementary Table S9: Statistical results corresponding to Figure 3E-F. Comparison of delta-band power during NREM sleep across light and dark pulses, averaged for CT12-18 under T2 (20-min bins) and CT14-21 under T7 (30-min bins) cycles and values obtained during corresponding time points under T24 cycle: results of ANOVAs (interaction between cycle, light condition and time for T2; interaction between cycle and time for T7) and HSD Tuckey's tests. Significant differences are in black whereas non-significant results are in grey. Grey compartments represent dark pulses.

Cycle x light condition x time			Delta-band power (Variable)
T2	effect of cycle		$F_{(2,16)} = 0.72$, $p = 0.50$<br>$F_{(1,8)} = 5.42$, $p < 0.05$#
	Subj. Day CT0-12	0-60 min	
		0-60 min	
			$F_{(2,16)} = 2.73$, $p = 0,10$
	Late subj. night CT18-24	0-60 min	
		0-60 min	
T7			$F_{(6,36)} = 4.37$, $p < 0.01$
	Subj. Day CT0-12	0-30 min	$p < 0.001$
		30-60 min	$p = 0.15$
		60-90 min	$p = 0.05$
		90-120 min	$p = 1.00$
		120-150 min	$p = 0.66$
		150-180 min	$p = 1.00$
		180-210 min	$p = 1.00$
		0-30 min	$p = 1.00$
		30-60 min	$p < 0.05$
		60-90 min	$p = 0.11$
		90-120 min	$p = 0.22$
		120-150 min	$p = 0.06$
		150-180 min	$p = 0.44$
		180-210 min	$p = 0.89$

Supplementary Table S10: Statistical results corresponding to Supplementary Figure S3G-I. Comparison of delta-band power during NREM sleep across light and dark pulses, averaged for subjective day (CT0-12) under T2 (20-min bins) or T7 (30-min bins) cycles, and for late subjective night under T2 cycle (CT18-24) and values obtained during corresponding time points under T24 cycle: results of ANOVAs (interaction between cycle, light condition and time, and, if non-significant, # effect of cycle) and HSD Tuckey's tests. Significant differences are in black whereas non-significant results are in grey. Grey compartments represent dark pulses.
